# A High-Temporal Resolution Technology for Dynamic Proteomic Analysis Based on ^35^S Labeling

**DOI:** 10.1371/journal.pone.0002991

**Published:** 2008-08-20

**Authors:** Zhao Zhang, Jian Chen, Fuzheng Guo, Liren He, Yizhou Wu, Changqing Zeng, Xueyuan Xiao, Dacheng He

**Affiliations:** Universities' Confederated Institute of Proteomics, Key laboratory for Cell Proliferation and Regulation Biology Ministry of Education, Beijing Normal University, Beijing, People's Republic of China; Deutsches Krebsforschungszentrum, Germany

## Abstract

As more and more research efforts have been attracted to dynamic or differential proteomics, a method with high temporal resolution and high throughput is required. In present study, a ^35^S *in vivo* Labeling Analysis for Dynamic Proteomics (SiLAD) was designed and tested by analyzing the dynamic proteome changes in the highly synchronized A549 cells, as well as in the rat liver 2/3 partial hepatectomy surgery. The results validated that SiLAD technique, in combination with 2-Dimensional Electrophoresis, provided a highly sensitivity method to illustrate the non-disturbed endogenous proteins dynamic changes with a good temporal resolution and high signal/noise ratio. A significant number of differential proteins can be discovered or re-categorized by this technique. Another unique feature of SiLAD is its capability of quantifying the rate of protein expression, which reflects the cellular physiological turn points more effectively. Finally, the prescribed SiLAD proteome snapshot pattern could be potentially used as an exclusive symbol for characterizing each stage in well regulated biological processes.

## Introduction

To decipher the intricate cellular activities more effectively at protein level, more and more researches have been focused on dynamic proteomics [1]–[6] or differential proteomics [7]–[9]. The dynamic change of protein quantity is the final outcome of some perplexed dynamic metabolic processes, such as protein expression, degradation, phosphorylation or exportation. However, most currently used technologies are targeted at the existed proteins in the cells, tissues and organs. The dynamic changes reflected by these methods are just at the total amount level, not at the expressed level, let alone other metabolic levels, despite the term “differential expressed proteome” has been used in most cases.

**Figure 1:**
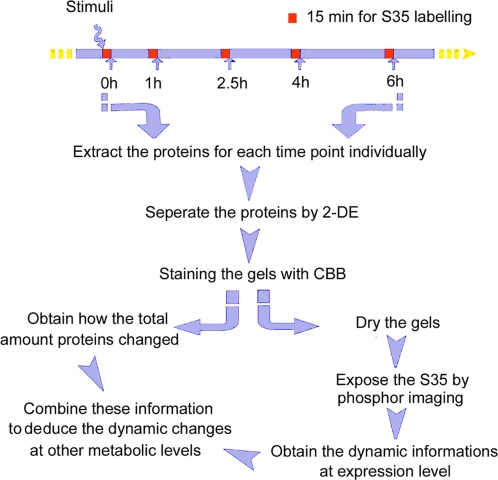
Flow Chart of SiLAD technology.

In this study, a novel method to examine the dynamic proteome expression changes based on ^35^S metabolic pulse labeling was proposed (Figure 1). 2-Dimensional Electrophoresis (2-DE) was employed in this methodology to detect the protein changes, since it is still the best technology to directly visualize the largest number of proteins simultaneously and separately, thus allowing us to detect most of the differential changing proteins before the Mass Spectrometry (MS/MS) identification. Yet, the selected differential proteins still could be identified by MS/MS. The auto-radiography of the ^35^S short pulse labeled gel provides the information about protein newly expressed without interference from the former existed amount. Using ^35^S to pulse label several time points in a biological process is, for the first time, allowing us to chase the proteome expression changes at the second order derivate level. The first order derivative level of protein amount change (S_p_) in the time course T is V_p,_ the changing velocity of protein amount. And
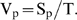



Changing of the V_p_ was measured by acceleration (A_p_), *i.e.* the second order derivative of protein amount change with respect to time. And
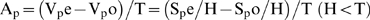



V_p_e means the ending velocity for a defined process,

V_p_o stands for the original velocity,

H means the time span we chosen to measure the V_p_e and V_p_o,

S_p_e and S_p_o stands for the protein changing amount in the time span H, at the ending and beginning of the process respectively.

(In our experiment, S_p_ was measured by ^35^S incorporation, H was 15 minutes and T was the time span between the two adjacent time points.)

The change of protein synthesis velocity, in stead of the change of the protein amount, is certainly representing the cellular functional turn point more directly and efficiently. This merit particularly fits the need of high throughput of proteomic research.

We have been applying this methodology to investigate the cell cycle progress within both serum starved A549 cells and rat liver hepatectomy model. Five time points were chosen in the process to pulse labeling the cells, with a labeling time of 15 min at each time point (Figure 1). Two kinds of 2-DE images were obtained sequentially: Coomassie Brilliant Blue-R 350 (CBB) staining images and the phosphor-images (PH-I). The dynamic changes at different levels for each protein spot can be deduced by combining the information supplied by these two detecting methods (Figure 1).

## Results

### Pulse labeling (15 min) provides an informative counter-profile with CBB staining

**Figure 2:**
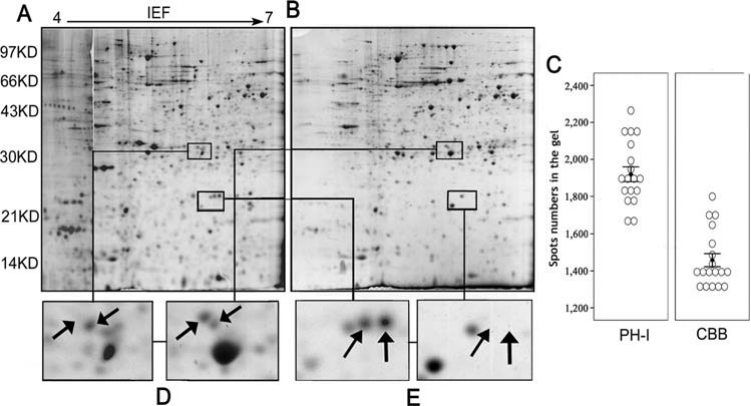
Evaluation of the developed ^35^S labeling protocol. (A) Phosphor-image of one 2-D gel of A549 cell at 1 hr time point. Labeling time was 15 min. pH-range 4–7 iso-electric focusing strips were used to separate protein in the first dimension and 12.5% polyacrylamide gels in the second dimension. (B) The CBB staining image for the same gel with A. (C) Statistical analysis of the spots numbers shown in the two kinds of images. PH-I stands for the images were got from Phosphor-imaging, while CBB means the CBB staining. The 18 gray circles in each block denote the spots numbers of 18 corresponding images (each time running at least 6 gels, 5gels for 5 time points and another gel for the proteins extracted from asynchronous cells as a control, repeated 3 times). Black error bars represent average spots numbers±s.e.m. for each kind of image. This chart was made by SPSS13. (D) Zoom in view of two spots, the spot labeled by upward arrow is protein PGDH, while the spot pointed with downward arrow is PRDX4. (E) Zoom in view of two spots only shown in the phosphor-image.

**Figure 3:**
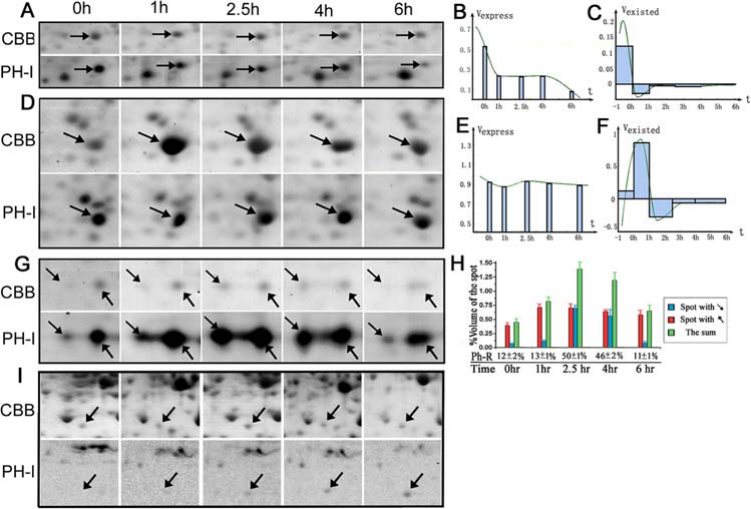
Dynamic metabolized changes of the proteins. (A, D, G and I) Zoom in view of the spots dynamic changes at different time points. Panel A, D, and G show three differential changed proteins in A549 model. The differential protein shown in panel I was from rat liver hepatectomy model. PH-I stands for the images were got from Phosphor-imaging, while CBB means the CBB staining. (B and E) Shown the dynamic expression changes of the spots labeled in panel A and D, respectively. The x axis is time (t), and the y axis is the protein synthesizing speed (v_expressed_), and the area of each rectangle means the total amount of protein synthesized during each 15 minutes interval (s_expressed_). The midpoint of each rectangle's top edge was used to interpolate these five points with piecewise cubic Hermite polynomial, as shown in the diagram as the green line. (C and F) Shown the dynamic total amount changes of the spots labeled in A and D. The x axis also is time (t), and the y axis is the total protein metabolized speed (v_existed_), consequently the area of each rectangle means the total amount of protein (s_existed_) variation during each time interval. This green line indicates the function of the variation speed of the total amount of existed protein (s_existed_) with respect to time (t), during these six hours. (H) Presence %Volumes changes of the protein labeled in chart G. Ph-R denotes phosphorylation rate, *i.e.* the percentage of phosphorylated proteins accounted for the total sum in each time point. The value of PH-R represents mean±s.e.m. for 3 independent experiments. Error bars represent mean±s.e.m., n = 3. Chart B, C, E and F were made by the software MatLab, and chart H was made by SPSS13.

All 2D gels were firstly stained with CBB. After drying, phosphor-imaging was used to quantify the ^35^S incorporation for the newly expressed proteins (Figure 1, methods). For all the 2D gels, two types of images were collected: by phosphor-imaging (Figure 2A), and CBB staining (Figure 2B). Spot-count in all the images was detected by Image Master Software followed by the same parameters. For A549 cell model, there were 1,929±40 spots in phosphor-images, compared with 1,461±36 spots in CBB staining images (Figure 2C), with a P-value in the Paired T-test of 5.15E-29 (n = 18), suggesting a higher sensitivity of PH-I over CBB detection. Figure 2A showed an example of phosphor-images, 2,264 spots were detected, while 1,799 spots detected in the CBB staining image (Figure 2B) for the same gel. There are 1651 spots matched in the two images, so the number of spot exclusively shown in phosphor-image is 613, account for 25.4% in the total 2,412 spots detected in this gel, compared with only 6.1% spots exclusively shown in the CBB staining. For the matched counter-spots, they exactly have the same position in the two kinds of images, thus greatly facilitate the following comparison, as shown in any zoom-in view of the 2-DE map (Figure 2 and 3). The 613 (25.4%) spots, *e.g.* the two spots labeled in Figure 2E, which can only be detected in phosphor-images, even the labeling time was only 15 min, reflecting the higher sensitivity of ^S^35 labeling method.

The protein spots that were only detected in CBB staining images, as pointed with the upward arrow in Figure 2D, representing they are either not contain Methionine and Cysteine, or they were expressed none or too little during the labeling period. An additional experiment was performed with 6 hours continuous labeling to distinguish all high/low sulfur containing proteins (data not shown). As an example, the spot pointed with the upward arrow in Figure 2D, with MS/MS identification, is the protein Hydroxyprostagladin Dehydrogenase (PGDH) which contains 11 Methionine and Cysteine, compared with only 5 Methionine and Cysteine in peroxiredoxin4 (PRDX4), pointed with the downward arrow. In this particular case, the spot of PRDX4 (contains less Methionine and Cysteine), but not PGDH, was shown clearly in the phosphor-image, indicating that PGDH was expressed at very low level or even not expressed at all in this process. This contrast between PH-I and CBB measurements shows another merit of the 35S metabolic labeling, high signal/noise ratio, just reflecting the protein newly expressed within the 15 min and without the interference from the former existed amount.

### High temporal-resolution to detect the dynamic protein expression changes

^35^S was used to pulse label the five time points in the chosen process, 15 min for each time point. A time series 2-DE maps for the protein were obtained (Figure 3A, 3D, 3G and 3I). The %volumes (the ratio between the volume of the spot and the sum of all the spots volume in the gel) of the 5 spots (from the same protein for the 5 time points) were calculated by the Image Master software which can withdrawn background automatically, and then 5 numerical values for the %volumes of these 5 spots were supplied by this software. Two kinds of temporal-resolution were acquired. One temporal resolution, by judging the five %volumes of the CBB spots, was how much the total amount of the same protein had been accumulated during the time span between each two adjacent time points (the traditional temporal-resolution, like non-labeled methods can obtained). The other temporal-resolution we obtained from the ^35^S exposed spots was about how much of the same protein was relatively expressed within each 15 min at different time points. As shown in the Figure 3A, comparison of the adjacent %volumes at the 4 and 6 hr time points revealed that the expression of the protein was decreased sharply over the period of two hours. As shown in the current experimental design, combining these temporal resolutions from all time points will enable the quantification of protein dynamic expression changes over the examined duration.

The %Volume of each spot is the reflection of the relative amount for the spot shown in the gels. Based on the phosphor-imaging data, at the same labeling duration, 15 min, %Volumes (the mean of three times repeated) at the five time points approximated the protein synthesizing velosity (v_expressed_). Interpolated v_expressed_ of the five points with piecewise cubic Hermite polynomial provided the function of protein synthesizing speed (v_expressed_) with respect to time (t). Its first order derivative is also the acceleration (a_expressed_), *i.e.* the second order derivative of total amount of synthesized protein (s_expressed_) with respect to time (t). As shown in Figure 3B, v_expressed_ of this protein was at its top level in the first time point, and then decreased steadily; and from the beginning of the first hour, v_expressed_ reached a constant level, and lasted till the fourth hour; then, v_expressed_ begins to decrease again and lasted till the end. For the %Volume data of existed proteins' total amount (s_existed_), which is from the CBB staining image, we interpolate them with piecewise polynomial of the cubic spline, and then take its first order derivative, which approximately indicates the function of the variation speed (v_existed_) of the total amount of existed protein (s_existed_) with respect to time (t), during these six hours. As shown in Figure 3C, the decreasing speed (v_existed_) of total protein begun to increase and later decline during the first hour and finally came to a stable situation with little oscillation, which lasted till the end. This result therefore is leading to a conclusion that the expression regulation of these proteins was drastically turned at these points, therefore these proteins may play a role in the cellular event occurred in this particular moment, and the low level expression maybe needed for the idle physiology activities.

In summary, ^35^S pulse labeling produces two kinds of temporal resolution to analyze the protein expression dynamic changes, one is for the labeling 15 min, and the other one is for the time span between each two time points. What is more, the high temporal resolution of this method enable it to display the function of the amount of newly synthesized protein and total existed protein with respect to time, as well as its second order derivative, *i.e.* the changes of the velocity. And it is the turn point of the velocity really to delegate the important key-point of cellular biological activity changes in the corresponding process.

### Digging out the protein new dynamic information hidden in traditional method

In A549 cell model, 164 dynamic changed spots (%volume have two times differential displayed in any two time points in CBB image or PH-I image or both) were detected and only 43 among them have the same change trend in both expression and total amount level. As for rat liver regeneration model, the proportion of the spot have the same change trend in the two levels is 49/124. These SiLAD data adequately demonstrated that the protein dynamic changes have huge differences between expression level and total amount level. The spot shown by Figure 3D, identified as Phosphoglycerate kinase 1 (PGK1), is an example to serve this principle. With traditional method, CBB staining provides the conclusion that the expression of this particular protein was increased sharply and attained its peak level at the one hour time point. But the ^35^S labeling data uncovered that the expression of this protein was at a constant level in the dynamic process, as shown in Figure 3E; In the opposite, the spot from liver hepatectomy model, was shown by traditional CBB measurement as a non-differential protein when the data from CBB staining profiles were just considered, but the ^35^S profiles showed this protein was not expressed before the liver surgery, but its synthesis speed was clearly increased after the hepatectomy surgery (Figure 3I). By this way, 30 new differential spots were discovered in liver regeneration model, which were hidden in the traditional analysis and more than 60% of the CBB defined dynamic changed proteins were under re-categorization (traditional non-differential proteins defined as differential at the expression level or vice versa).

SiLAD has also been successfully used to mine the information for some proteins' dynamic degradation or exportation. The above mentioned protein PGK1, is mainly carries out a function in glycolysis and DNA replication in the cell [10], [11]. The total amount of this protein (s_existed_) was significantly raised up in this studied process (Figure 3F), but the v_expressed_ had been unchanged (Figure 3E), suggesting that the dynamic changes of the total existed protein is not caused by its synthesis speed, but due to some related metabolism process such as protein degradation or exportation (for secrete proteins). At the first time point, the synthesis and degradation of PGK1 was in a balance. Upon serum stimulation, however, the degradation of this protein was declined sharply and made more proteins accumulated at the 1 hr time point. But as the cell cycle progress keeps going, the degradation of this protein was speed up and leads to the proteins presented in the images became less and less, even though its expression velocity had been relatively stable. In this case of protein PGK1, the possible contribution of protein exportation cannot be completely ruled out, since it was reportedly to be secreted by tumor cells as a disulphide reductase [11].

SiLAD data also provides some valuable information about protein PTM (Post Translational Modification) dynamics, for example phosphorylation changes, measuring how much newly expressed protein is phosphorylated in the 15 min. According to the special pattern of protein phosphorylated isoforms in the 2D gel and MS/MS identification [12], some spots in the almost horizontal positions identified as identical protein represent the different phosphorylated isoforms. In Figure 3G, the two spots were both identified as protein translational-controlled tumor protein (TCTP). The spot labeled with downward arrow is the phosphorylated form and the spot pointed with upward arrow is none or less phosphorylated. In the labeling 15min of the 0 hr time point, there was little newly synthesized proteins shown in the phosphorylated spot (PH-I image). At the early stage of progress, the phosphorylated proportion of the newly synthesized proteins kept very low, though the total newly synthesized proteins was increased. But in the time span from 2.5 hr to 4 hr, the phosphorylation was in such a high level that made almost half of the newly synthesized proteins, which successfully be labeled in the 15 min, be phosphorylated. And as the process keeps going, the phosphorylation of this protein was decreased significantly and got back to its original level at the 6 hr (Figure 3H). These dynamic changes implied that the phosphorylation of TCTP from 2.5 hr to 4 hr might be significant to guarantee this process running smoothly. These results was in consistent with the early reports that TCTP dynamic phosphorylation is necessary for certain imperative cell cycle regulation events [13], [14].

In summary, as we showed in this paper, adding the new information provided by SiLAD, all differential proteins will be under a re-category, and their dynamic curve can be described more precisely, the novel information about protein degradation, modification and so on gives us a much more comprehensive picture of the proteins in a cellular context.

### SiLAD profiles could serve as exclusive bar code for the specific temporal position in a dynamic process, just as 5-bromo 2-deoxyuridine (BrdU) labeling for S phase

**Figure 4:**
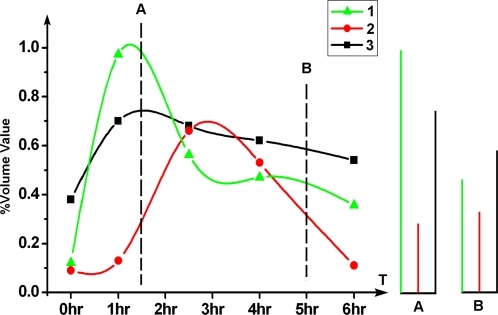
SiLAD profiles are served to characterize the specific state of the cell cycle progress. Curve 1 was from the CBB spot in Figure 3D. Curve 2 was from the spot in Figure 3G PH-I image labeled with downward arrow. Curve 3 was from the same image with Curve 2, but was the spot labeled with upward arrow. The right panels A and B were the defined bar codes for the two time points labeled with spotted line A and B in the left panel. The curve was made by the software Origin6.

The comparison of the two kinds of time series 2D profiles obtained by the two visualizing methods (CBB and PH-I), provided dynamic curves for a large number of proteins, with not only their existing amounts but also their synthesis activity. A combination pattern containing a couple of (as few as three) chosen differential proteins could serve as exclusive “bar code” for every time point in a well regulated cellular process. Figure 4 shows the specific bar codes for the different moments in A549 model containing the existing amount and synthesis intensity of three spots (one spot from Figure 3D CBB image and two spots from Figure 3G PH-I image). After our carefully compared, as the curve knots in this figure illustrated, each time points have their exclusive bar code derived from the %volumes of these three spots. After appropriate simulate the spot %volume values of the five time points to form these three curves (Figure 4), a bar code can set for any specific moment besides the original five time points set for the measuring. As the examples shown, for the two time points marked by A and B spotted line in the left panel in Figure 4, there are two respective unique bar code patterns shown in the A and B panels in this Figure. In our experiments, there are always more than enough differential spots ready provided for choice to construct efficient bar code, 164 differential spots in A549 cell line model and 124 differential spots in rat liver hepatectomy model. Of course, a careful selection is still essential to optimize the bar code.

**Figure 5:**
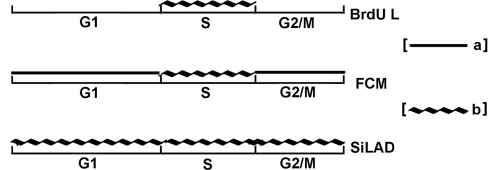
Comparison among BrdU labeling, Flow Cytometry and SiLAD to check cell cycle stage. Black line a means only to determine the cell cycle phase roughly. Curve line b means detecting the specific time point exactly. BrdU L is stands for the BrdU labeling and FCM is Flow Cytometry.

In this study, the SiLAD bar code was compared with two alternative methods, BrdU labeling and Flow Cytometry, which are also used to provide the temporal coordinate when a continuous bio-process is investigated. With BrdU labeling, an experienced researcher can easily discriminate four or five stages of S phase in cell cycle based on the characteristic spatial patterns of nuclear staining [15]. Flow cytometry provides the information to distinguish the whole G1 or G2/M phase and different stages within S phase. SiLAD bar code, in stead, can be used to determine all the temporal positions throughout the whole biological procession, yet with much higher resolution. This comparison was summed in the Figure 5. However, this SiLAD coordinate system has to be defined individually for each desired bio-process prior to use.

## Discussion

Currently, a huge amount of data in differential proteomics field has been generated to compare the “differential expression” of proteins. Unfortunately, most of them were dealing with the total existed proteins in stead of their “expression”. What is more, in Differential Proteomics sphere, the scientist always confused Differential Proteomics with Differential Expressed Proteomics. The changes of the proteins they obtained were always reckoned to be at expression level, which is at total amount level in fact. In the present study, SiLAD, a high temporal resolution technology to deal with the real “differential expression” of proteome and related broader dynamic changing was developed. And based on our data, this method could reflect the dynamic changes of cell physiological activities more efficiently.

**Figure 6:**
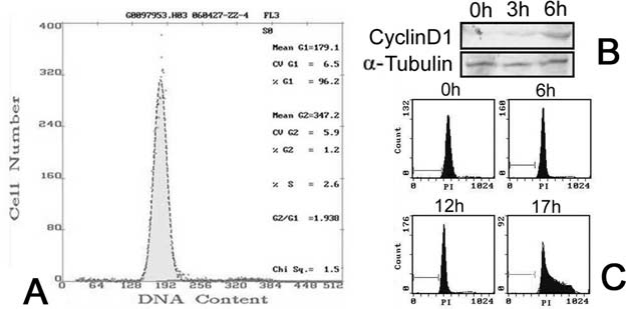
Evaluation the synchronized efficiency of serum starved A549 cells. (A) FACS analysis of A549 cells synchronization by serum starvation for 48 hr. (B) Western blot analysis of expression of CyclinD1 at the indicate time lengths after serum re-stimulation. (C) FACS to detect the cell cycle progress after serum re-stimulation.

Protein dynamic changing is a continuous process in single cell. To investigate the protein dynamics of a well regulated process, three kinds of models are seemly suitable: a single cell; highly synchronized cell population and a biological system with a natural or external starting point such as an infection or a treatment. In this study, a synchronized cell line and an operation induced liver regeneration model were employed. For *in vitro* cell line, six cell lines had been screened. Among them, A549 cell line successfully reached 94% synchronization under serum starvation (Figure 6A). And according to the former reports, it is a commonly accepted model to study the early cell cycle phase transition [16], [17]. To verify the cell cycle progress was going normally after the serum deprivation, we had also detected the expression status of CyclinD1 protein (Figure 6B), a marker that reportedly can be detected in early G1 phase [18]–[20]. At the same time, rat liver regeneration was used as an *in vivo* model. Cell division is rarely seen in hepatocytes in normal adult liver. After partial hepatectomy, approximately 95% of the hepatic rapidly enter the cell cycle [21]–[23]. Compared with A549 cells, a smaller spot number and lower signal/noise ratio were obtained from ^35^S 2D profile in liver regeneration model, most possibly due to the lower ^35^S labeling efficiency *in vivo* and the existence of other minor cell types in the liver. 15 minutes pulse labeling was determined after a series of optimization to ensure the high temporary resolution yet avoid the potential damage to cells, such as inhibiting cell proliferation and inducing DNA fragmentation [24].

This novel technique detects and measures the newly expressed proteins separately from the existing proteins, therefore brings several advantages. Firstly, it better fits the common goal of differential expressed proteins researches. As the labeling time is only 15 min, it is not only leading to a higher temporal resolution, more importantly, it provides the velocity of the synthesis of desired proteins, *i.e.* how actively expressed were these proteins at the present moment, which makes more sense than the total amount of proteins does in describing and understanding the dynamic process, particularly on the physiology turn points. For the same reason, many new differential proteins could be discovered and all traditionally defined “differential proteins” may need to be re-categorized. Secondly, since the interference from the accumulated proteins, which usually in a much higher amount over the newly synthesized proteins, is excluded by our method, the sensitivity and signal/noise ratio of SiLAD is significantly improved. Assuming the total protein amount at time point one was 100+1 (100 for the already accumulated proteins and 1 for the newly synthesized), at time point two is 100+5, then if judged from the already existed amount by CBB, the difference is (105−101)/101 = 104%, which would be considered as “no change”. However, when judged by PH-I, the difference is (5−1)/1 = 400%, the change is very significant. In the present study, the protein shown in Figure 3I represented such a situation. Thirdly, compared with some newly developed method that also employed isotope, such as ICAT and SILAC [7], [9], SiLAD omits the tough work to find the potential different proteins from thousands of proteins one by one by MS/MS. It visualized all differential proteins simultaneously so long as they can present in 2-DE profile and therefore has the merit to fit the urgent need of high throughput proteomics research, particularly the bio-marker discovery research, which is an emerging field currently. Fourthly, based on the SiLAD technique, an exclusive bar code and a temporary coordinate system could be constructed to benefit future research works, which concern the temporary position in a dynamic process.

In summary, the SiLAD technique as presented in this study could be applied to a very broad research area far beyond cell cycle or biomarker subjects. Moreover, it is easy and fast to perform, no special or extra equipments are required.

## Materials and Methods

### A549 cell cultures, cell cycle synchronization and flow cytometry (FCM) analysis

A549 lung carcinoma cells were from American Type Culture Collection and were cultured in RPMI 1640 medium (Gibco-BRL) containing 10% fetal bovine serum (FBS) (Gibco-BRL) and maintained at 37°C in a humidified atmosphere with 5% CO_2_. Cells were synchronized by serum deprivation for 48 hr. After starvation, cells were stimulated in the medium with 10% FBS to initiating a new cell cycle .

To analyze DNA content, cells were trypsinized, washed twice with PBS, then were suspend in 75% chilled ethanol and kept at 4°C over night. Fixed cells were treated with 50 µg/ml RNaseA in PBS at 37°C for 30 min, and then stained with 50 µg/ml Propidium iodide (Sigma)on ice for 30 min. DNA content was determined by flow cytometry using FACS-can (EPICS®XL, COUCTER ), and data were analyzed with the software System 2.

### ^35^S pulse labeling the A549 cells and protein extraction

30 min prior to the designated time points (0 hr, 1 hr, 2.5 hr, 4 hr, 6 hr) post-stimulation, cells were washed twice with Met&Cys-free 1640 medium (Sigma) and maintained in this medium for 10 min. Then the cells were labeled with ^35^S-Met and ^35^S-Cys by adding NEG-722 EASYTAG^TM^ EXPRESS PROTEIN LABELING MIX (PerkinElmer) to the Met&Cys-free medium with the final concentration 80 μCi/10^6^cells. The pulse labeling process was maintained for 15 min and then terminated by rinsing the cells twice with normal 1640 medium.

After labeling, the cells were trypsinized immediately, washed them twice with cold washing buffer (250 mM Sorbitol,Amresco, 10 mM Tris-HCl, Amresco, pH7.4) and centrifuged (1000 rpm for 5 min). The cell pellet was suspended in a lysis buffer containing 6 M urea, 2 M thiourea, 4% CHAPS, 1%DTT, 0.5% IPG buffer pH 4–7 (Amersham Biosciences). The suspension was placed on ice for 1 hr with gentle vortex every 15 min, followed by centrifugation at 15000 rpm for 30 min twice. Protein concentration was determined by the Bradford method.

### Rat liver 2/3 hepatectomy surgery and^ 35^S pulse labeling

The protocol for rat liver 2/3 hepatectomy has described previously [23]. Briefly, Adult male Sprague-Dawley rats (220–280 g) were obtained from the Experimental Animal House at Peking University. After overnight fasting with free access to water, the rats were subjected to an operation removing 70% of their liver at 8:00–10:00 A.M. The proteome dynamic changes after surgery were monitored at 0 hr, 1 hr, 2.5 hr, 4 hr and 6 hr following liver hepatectomy by ^35^S metabolic labeling (peritoneal injection of 50 µl NEG-722 EASYTAG^TM^ EXPRESS PROTEIN LABELING MIX, PerkinElmer, for each rat). After labeling 15 min for each time points, rats were sacrificed to extract the liver proteins.

### 2-DE, gel drying, exposing and scanning the phosphor screen, and image analysis

The 2-DE was essentially carried out as described in the 2-DE handbook from Amersham Corporation by using the Amersham's Ettan IPGphor and Ettan DALT Six Systems. Briefly, each sample of 0.75 mg protein (different time points) was loaded on immobilized 18 cm, pH 4–7 strip (GE Healthcare). After rehydration at 30 V for 12 hr, IEF was carried out using the following conditions: 100 V for 1 hr, 500 V for 1 hr, 1000 V for 1 hr, 8000 V gradient for 30 min and 8000 V for 60000 Vhr. Following IEF, the gel strip was first equilibrating for 15 min in the equilibration buffer containing 1% DTT (Sigma). Then the gel strip was equilibrated for another 15 min in the same equilibration buffer, except that DTT was replaced with 2.5% iodoacetamide (Sigma). The second dimensional SDS-PAGE was performed in 12% acrylamide gels. The gels were stained using CBB R350 (Amersham Biosciences). Electric images of the gels were obtained using the MagicScan densitometer using the LabScan software (Amersham Biosciences) at the same scanning parameters. The Tiff format images were used for the image analysis.

After dyeing with CBB-R350, the gels were soaked over night in the buffer containing 20% Methanol and 3% Glycerol. The gels were dried with the Gel-Dryer (MODEL583 GEL DRYER, BIO-RAD). Exposing and scanning the phosphor screen was carried out as described in the manual book from the Packard Corporation by using the Cyclone Storage Phosphor System, with an exposing time of 5 days. Two images for each gel were acquired, one is from the Coomassie Blue Staining, and the other one is from the phosphor-imaging. Image analysis was carried out with ImageMaster 2-D Platinum software (Amersham Biosciences). Briefly, protein spots were automatically detected first and then refined manually; subsequently the spots in the gels were normalized by using of relative volume (%Vol), which is the ratio between its volume and the sum of all the spots volume in the gel were calculated and used for quantitative comparison and then matched.

### Protein identification by Mass Spectrometry

To identify the interested proteins, each spot was excised from the gels, transferred to the 0.5-mL siliconized Eppendorf tube, trypsin digested and the resulting fragments were analyzed on ESI-QTOF-MS. Briefly, the excised spots were washed three times with Milli-Q water, and then de-stained with 25 mM NH_4_HCO_3_/50% ACN until the gel pieces were completely de-stained. Finally, the gel pieces were washed with 100% ACN for 5 min, and then dried. The dried gel pieces were incubated in 10 µl trypsin solution (concentration: 10 ng/ml) at 37°C overnight. The resultant peptide fragments were analyzed with Qstar Pulser I Quadrupole TOF-MS (Applied Biosystems/MDS Sciex, Canada). The protein identification was determined by MS/MS fragment ion using MASCOT software through searching the Swiss-Prot database with the search parameters: variable modifications are carbamidomethyl/oxidation, allow up to 2 missed cleavages, 100 ppm for peptide tolerance and 0.1 Da for MS/MS tolerance.

### Western blotting

In brief, samples of 60 µg were separated into 12% SDS-PAGE gels and the proteins were transferred onto Immobilon-P membranes (Millipore) for 2 h at 60 V. Subsequently, the membranes were blocked with TBS containing 3% BSA for 1 hr at room temperature. The membranes were then incubated in primary antibody (MBL, 1∶500 dilutions) over night at 4°C. After incubation, the membranes were exposed to secondary antibody (1∶1000 dilutions) and developed by nitro blue tetrazolium/5-bromo-4-chloro-3-indolyl phosphate (NBT/BCIP). The digital image was obtained by scanning the membrane on the densitometer.
